# Identification of the Minimum Combination of Serum microRNAs to Predict the Recurrence of Colorectal Cancer Cases

**DOI:** 10.1245/s10434-022-12355-w

**Published:** 2022-09-29

**Authors:** Yukihiro Yoshikawa, Mitsuko Fukunaga, Junichi Takahashi, Dai Shimizu, Takaaki Masuda, Tsunekazu Mizushima, Kazutaka Yamada, Masaki Mori, Hidetoshi Eguchi, Yuichiro Doki, Takahiro Ochiya, Koshi Mimori

**Affiliations:** 1 Department of Surgery, Kyushu University Beppu Hospital, Beppu, Oita Japan; 2 Department of Gastroenterological Surgery, Graduate School of Medicine, Osaka University, Suita, Osaka Japan; 3 Coloproctology Center Takano Hospital, Kumamoto, Kumamoto Japan; 4 Department of Surgery and Science, Graduate School of Medical Sciences, Kyushu University, Fukuoka, Japan; 5 Division of Molecular and Cellular Medicine, National Cancer Center Research Institute, Tokyo, Japan

## Abstract

**Background:**

Serum microRNAs (miRNAs) have been recognized as potential stable biomarkers for various types of cancer. Considering the clinical applications, there are certain critical requirements, such as minimizing the number of miRNAs, reproducibility in a longitudinal clinical course, and superiority to conventional tumor markers, such as carcinoembryonic antigen (CEA) and carbohydrate antigen 19-9. This study aimed to identify serum miRNAs that indicate the recurrence of colorectal cancer (CRC), surpassing inter-tumor heterogeneity.

**Methods:**

We conducted an analysis of 434 serum samples from 91 patients with CRC and 71 healthy subjects. miRNAs were obtained from Toray Co., Ltd, and miRNA profiles were analyzed using a three-step approach. miRNAs that were highly expressed in patients with CRC than in the healthy controls in the screening phase, and those that were highly expressed in the preoperative samples than in the 1-month postoperative samples in the discovery phase, were extracted. In the validation phase, the extracted miRNAs were evaluated in 323 perioperative samples, in chronological order.

**Results:**

A total of 12 miRNAs (miR-25-3p, miR-451a, miR-1246, miR-1268b, miR-2392, miR-4480, miR-4648, miR-4732-5p, miR-4736, miR-6131, miR-6776-5p, and miR-6851-5p) were significantly concordant with the clinical findings of tumor recurrence, however their ability to function as biomarkers was comparable with CEA. In contrast, the combination of miR-1246, miR-1268b, and miR-4648 demonstrated a higher area under the curve (AUC) than CEA. These three miRNAs were upregulated in primary CRC tissues.

**Conclusion:**

We identified ideal combinatorial miRNAs to predict CRC recurrence.

**Supplementary Information:**

The online version contains supplementary material available at 10.1245/s10434-022-12355-w.

Colorectal cancer (CRC) is the third most common cancer and the second leading cause of cancer-related deaths worldwide, with an estimated 1.2 million new cases and half a million deaths each year.^1^ Even when primary lesions are resected with the intent to cure, relapse is common in patients with advanced CRC.^2^ Several studies have revealed that adjuvant therapies can dramatically improve the prognosis of patients with advanced CRC by eliminating undetectable remnant cancer.^3–5^ This finding suggests that early diagnosis of metastatic CRC and early therapeutic intervention can improve the clinical outcome of patients who experience relapse, and provide an opportunity for cure. Therefore, early diagnosis of CRC at a subclinical level is pivotal for metastatic CRC.

Current diagnostic imaging tools, such as contrast-enhanced tomography (CT), positron emission tomography CT (PET-CT), and magnetic resonance imaging (MRI), can facilitate the detection of CRC metastases;^6^ however, these modalities are of limited value due to their inability to identify early metastatic and recurrent lesions. Similar to the current screening tools for detecting CRC, including colonoscopy, occult blood testing, and plasma-based assays; each modality has disadvantages, including morbidity^7^ and low sensitivity and specificity.^8^ As peripheral blood can be non-invasively obtained and easily stored, detecting multiple serum biomarkers has become an alternative method for early diagnosis of CRC. Although carcinoembryonic antigen (CEA) and carbohydrate antigen (CA) 19-9 have been widely used for auxiliary CRC diagnoses and prognostic predictions,^9,10^ previous studies have reported that CEA and CA19-9 present a diagnostic sensitivity of <50%.^11,12^ This means that more than half of the patients with CRC will be misdiagnosed based on CEA or CA19-9 alone. Therefore, the identification of more useful serum biomarkers for detecting CRC is urgently needed to enable early diagnosis and timely treatment of CRC.

MicroRNAs (miRNAs), a class of small non-coding RNAs measuring 19–22 nucleotides in length, function as post-transcriptional regulators by directly cleaving target messenger RNAs or via translational repression.^13^ Many miRNAs have been reported to be associated with cancer development and have shown to be potential biomarkers in various cancers, including CRC.^14–25^ However, independent studies have failed to develop clinically actionable miRNA biomarkers to date. The genomic or transcriptomic diversity in advanced CRC may cause difficulties in identifying the preferred biomarker for recurrence.

In the current study, we conducted the following approaches. First, we proved that multiple miRNAs (12 miRNAs) could surpass the inter-tumor heterogeneity, indicating high sensitivity and specificity to detect recurrence among CRC cases. Second, we examined three independent cohorts, namely the screening, discovery, and validation phases for miRNA microarray assays, to consolidate reproducibility. Third, we implemented continuous sampling during longitudinal observation postoperatively to stabilize miRNA expression. We then determined the most optimal but minimized number of combinations of miRNAs for clinical use.

## Methods

### Clinical Samples

In total, 323 perioperative chronological serum samples were obtained from 71 stage II/III patients with CRC treated at the Coloproctology Center Takano Hospital between 2014 and 2016. A total of 40 serum samples and paired sets of preoperative and 1-month postoperative samples were obtained from 20 stage II/III patients with CRC treated at Osaka University Hospital between 2015 and 2017. Seventy-one serum samples were obtained from normal control (NC) patients who underwent a medical examination at the Usatakada Regional Adult Diseases Medical Examination Centre between 2014 and 2016, without severe medical history. Serum samples were stored at – 80 °C until use. Clinicopathological data were collected from medical records and written informed consent for the use of clinical samples and data were obtained from all patients. This study was approved by the Ethics Board of Kyushu University Hospital (No. 689-00). All methods were performed in accordance with the relevant guidelines and regulations.

### Three-Step Approach for Identifying Candidate Biomarkers

*Screening phase:* miRNA expression in the 91 preoperative samples from Coloproctology Center Takano Hospital and Osaka University Hospital was compared with that in the 71 NC samples. We extracted miRNAs that were significantly upregulated in the serum of patients with CRC compared with that of NCs.

*Discovery phase:* miRNA expression in paired preoperative and 1-month postoperative serum samples from 91 patients from the Coloproctology Center Takano Hospital and Osaka University Hospital were compared. We extracted miRNAs that were significantly upregulated before operation.

*Validation phase:* We analyzed 323 perioperative chronological serum samples from 71 patients at the Coloproctology Center Takano Hospital, and identified miRNAs whose expression decreased in the postoperative period when compared with the preoperative period in non-recurrence cases and re-elevated before recurrence in recurrence cases.

### MicroRNA (miRNA) Expression Microarray Analysis

Serum samples were collected and prepared based on the standard operating procedure of Early Detection Research Network at the National Cancer Institute (https://edrn.nci.nih.gov/). Total RNA was extracted from a 300 μL serum sample using a 3D-Gene^®^ RNA extraction reagent from a liquid sample kit (Toray Industries, Inc., Kanagawa, Japan). Comprehensive miRNA expression analysis was performed using a 3D-Gene^®^ miRNA labeling kit (Toray Industries, Inc.) and a 3D-Gene^®^ Human miRNA Oligo chip (Toray Industries, Inc.), which was designed to detect 2555 miRNA sequences registered in miRBase release 20 (http://www.mirbase.org/).

miRNAs were considered to be present if the corresponding microarray signal was greater than the (mean + 2 × standard deviation) signal of the negative controls, of which the top and bottom 5% ranked by signal intensity were removed. Once the miRNAs were detected, the mean signal of the negative controls from which the top and bottom 5% ranked by signal intensity were removed was subtracted from the miRNA signal. When the signal value was negative (or undetected) after background subtraction, the value was replaced by the lowest signal intensity on the microarray minus 0.1 on a base 2 logarithm scale. To normalize the signals across the different microarrays tested, miRNA expression profiles were quantile-normalized and subjected to the ComBat method to remove batch effects.

### miRNA Expression Data of Colon Cancer Tissue from a Public Database

miRNA expression data from GSE49246 (deposited in the Gene Expression Omnibus [GEO] repository: https://www.ncbi.nlm.nih.gov/gds),^22^ which included a total of 40 paired samples of colon cancer tissue and adjacent normal colon tissue, were used to predict miRNA origin.

### miRNA Target Prediction

The target genes of miR-1246, miR-1268b, and miR-4648 were predicted using TargetScan (http://www.targetscan.org/vert_72/) and miRDB (http://mirdb.org/miRDB/), which are commonly used to predict miRNA targets.

### Statistical Analysis

Serum miRNA expression profiles were compared between the two groups using the Mann–Whitney U test. We performed a receiver operating characteristic (ROC) curve analysis of CRC recurrence. The cut-off value was determined by the point on the ROC curve with the minimum distance from the left-upper corner of the unit square. Fisher’s linear discriminant analysis was performed, and diagnostic sensitivity, specificity, and accuracy were calculated. Sensitivity and specificity were compared using McNemar’s test. For algorithm development, discriminant functions were created using Fisher’s linear discriminant analysis. The resulting values of the discriminant functions were used to prepare the diagnostic index. In clinical samples, an index of not less than 0 was considered to indicate a tumor-bearing condition, and an index of less than 0 was considered to indicate a non-tumor-bearing condition. Statistical significance was set at *p* values <0.05.

All analyses were performed using R version 3.3.1 (The R Foundation for Statistical Computing, Vienna, Austria; http://www.R-project.org), MASS package version 7.3–30 and bee swarm package version 0.1.6, pROC package version 1.12.1, and corrplot version 0.84.

## Results

### Identification of Candidate Colorectal Cancer (CRC)-Associated miRNAs

**Fig. 1:**
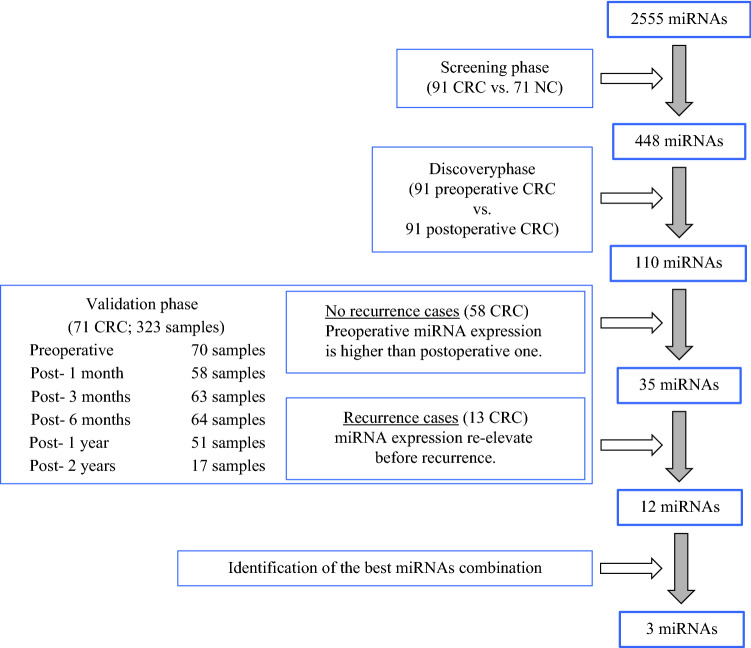
Study design flow chart. The study consisted of three sections: a screening phase for selecting candidate miRNAs as biomarkers for the recurrence of CRC; a discovery phase for investigating the candidate miRNAs; and a validation phase for constructing diagnostic models and confirming the optimal miRNAs. *miRNAs* microRNAs, *CRC* colorectal cancer, *NC* normal control

A total of 434 serum samples from 91 patients with stage II/III CRC, 323 perioperative chronological serum samples from 71 patients, 40 paired serum samples from 20 patients, and 71 NC subjects were analyzed in a three-step approach that included a screening phase, a discovery phase, and a validation phase. A flowchart of the experiment is illustrated in Fig. 1. Patients with CRC were diagnosed using colonoscopy at the Coloproctology Center Takano Hospital or Osaka University Hospital. NCs without a severe medical history underwent a medical examination at the Usatakada regional adult diseases medical examination. The demographics and clinical features of the study subjects are shown in electronic supplementary material (ESM) Table S1. The positive lymph node metastasis rate was 57.1%. Thirteen patients had recurrence; time to recurrence was 15.6 ± 7.4 months, recurrence location was the lung in ten cases and the liver in three cases. We performed an unsupervised two-way hierarchical clustering of miRNAs and samples, and confirmed that the miRNA expression levels and baseline characteristics were not associated (ESM Fig. S1). Staging was performed according to the 8th TNM classification.^26^ There were no associations between miRNA expression and age, sex, stage, region, or histology.

**Fig. 2:**
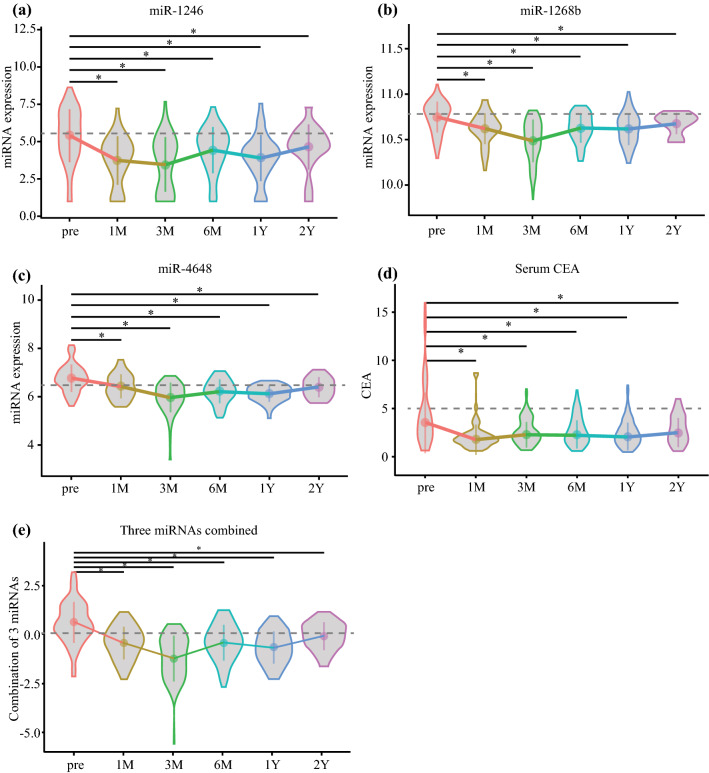
Timeline of three miRNA and combined miRNA expression levels in no-recurrence cases. **a** miR-1246; **b** miR-1268b; **c** miR-4648; **d** CEA; **e** three combined miRNAs. Horizontal dotted line indicates the cut-off value; and point and vertical line indicate the mean with 95% confidence interval. ^*^p < 0.05. *CEA* carcinoembryonic antigen, *M* month, *Y* year

**Fig. 3:**
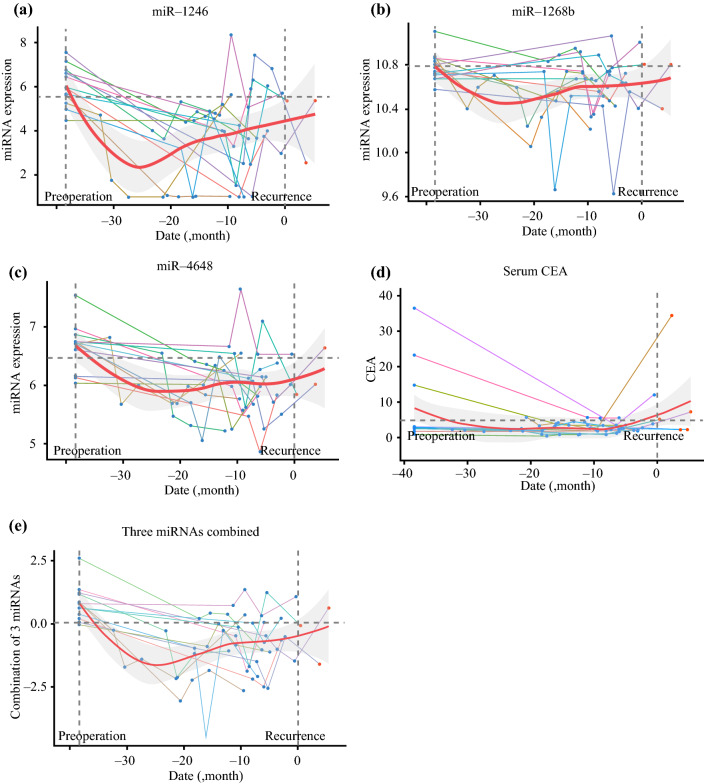
Timeline of three miRNA and combined miRNA expression levels in recurrence cases. **a** miR-1246; **b** miR-1268b; **c** miR-4648; **d** CEA; **e** three combined miRNAs. Horizontal dotted line indicates the cut-off value; left vertical dotted line indicates the preoperative point; right vertical dotted line indicates the recurrence point; line chart represents each patient’s expression level timeline; red line indicates Loess smoothing curve of the mean; and the grey area represents the 95% confidence interval. *miRNA* microRNA, *CEA* carcinoembryonic antigen

In the screening phase, to identify miRNA-based biomarkers for the recurrence of CRC, we compared miRNA expression between preoperative serum obtained from stage II/III patients and samples from healthy donors (normal control; NC). Of the 2555 miRNA probes analyzed by microarray analysis, 448 miRNAs were significantly upregulated in the serum of patients with CRC compared with that in the NCs. Next, in the discovery phase, we focused on serum miRNAs that were upregulated preoperatively as opposed to postoperatively. We extracted 110 miRNAs that were significantly more abundant in the preoperative samples than in the other samples. Finally, in the validation phase, we chronologically evaluated the expression levels in candidate serum. In the no-recurrence group (*n* = 58), the expression levels of 35 miRNAs were higher than that at any of the postoperative points, including 1 month, 3 months, 6 months, 1 year, and 2 years. Therefore, we expected to identify the re-elevation of miRNAs among those 35 miRNAs as an indicator of recurrence. In the recurrence group (*n* = 13), we found the re-elevation of 12 miRNAs (miR-25-3p, miR-451a, miR-1246, miR-1268b, miR-2392, miR-4480, miR-4648, miR-4732-5p, miR-4736, miR-6131, miR-6776-5p, and miR-6851-5p) before recurrence, and the 12 identified miRNAs should be invisible biomarkers indicating the recurrence of malignant cells. The timelines of the expression of the 12 identified miRNAs are shown in Fig. 2 and ESM Fig. S2 (no recurrence cases) and Fig. 3 and ESM Fig. S3 (recurrence cases).

Next, we performed ROC analysis of the recurrence of CRC using CRC-present samples (preoperative samples in all cases and postoperative samples after recurrence in recurrence cases) and CRC-absent samples (postoperative samples in no-recurrence cases). The area under the curve (AUC), sensitivity, and specificity are shown in Table 1. The sensitivity and specificity of CEA were 38.3% and 94.5%, respectively (cut-off value = 5.0 ng/mL), and the sensitivity and specificity of CA19-9 were 15.3% and 89.9%, respectively (cut-off value = 37.0 U/mL). Each single miRNA was inferior to CEA (Table 1).

**Table 1:** Twelve miRNAs identified as candidate biomarkers of the recurrence of CRC

	CEA	miR-25-3p	miR-451a	miR-1246	miR-1268b	miR-2392	miR-4480	miR-4648
AUC	0.725	0.737	0.709	0.749	0.745	0.778	0.698	0.763
Sensitivity	0.383	0.274	0.479	0.452	0.397	0.301	0.205	0.384
Specificity	0.945	0.902	0.902	0.912	0.931	0.902	0.902	0.902
*P*-value (vs. CEA)	–	0.792	0.792	0.571	0.717	0.281	0.685	0.446

	miR-4732-5p	miR-4736	miR-6131	miR-6776-5p	miR-6851-5p	3 miRNAs	4 miRNAs	5 miRNAs
AUC	0.702	0.787	0.725	0.733	0.706	0.821	0.829	0.835
Sensitivity	0.315	0.370	0.438	0.260	0.260	0.507	0.507	0.534
Specificity	0.917	0.907	0.912	0.902	0.902	0.902	0.902	0.902
*P*-value								
(vs. CEA)	0.727	0.128	0.949	0.838	0.695	0.0472	0.0226	0.0144

*miRNAs* microRNAs, *CRC* colorectal cancer, *CEA* carcinoembryonic antigen, *AUC* area under the curve

### Combinations of miRNAs as Biomarkers for the Recurrence of CRC

**Fig. 4:**
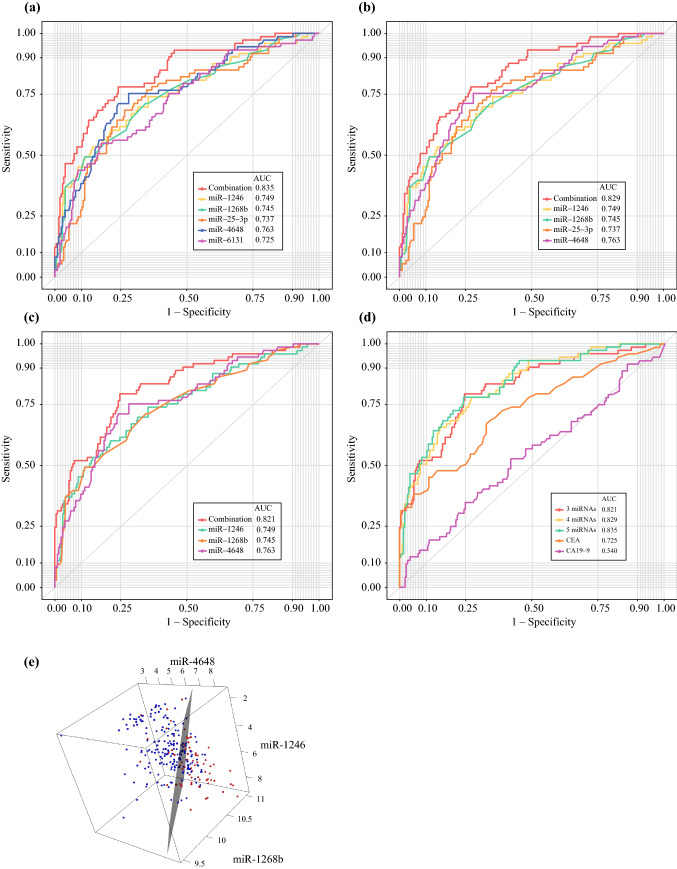
Analysis of the diagnostic index using the combinations and panel of miRNAs. **a** ROC curves for the combination of five miRNAs (miR-25-3p, miR-1246, miR-1268b, miR-4648, and miR-6131). **b** ROC curves for the combination of four miRNAs (miR-25-3p, miR-1246, miR-1268b, and miR-4648). **c** ROC curves for the combination of three miRNAs (miR-1246, miR-1268b and miR-4648). **d** Comparison of each combination, CEA, and CA19-9. **e** The cut-off line of the combination of three miRNAs in three dimensions: blue indicates no cancer; red indicates cancer. *miRNAs* microRNAs, *ROC* receiver operating characteristic, *CA19-9* carbohydrate antigen 19-9

Using Fisher’s linear discriminant analysis, we designed comprehensive discriminants with 1–5 miRNAs from the 12 identified miRNAs as biomarkers for the recurrence of CRC, as reducing the miRNA number can reduce the cost and complexity of the test. The analysis identified a combination of five miRNAs (miR-25-3p, miR-1246, miR-1268b, miR-4648, and miR-6131), four miRNAs (miR-25-3p, miR-1246, miR-1268b, and miR-4648), and three miRNAs (miR-1246, miR-1268b, and miR-4648) that showed much better discrimination than each single miRNA (Figs. 4a–c). The diagnostic index was calculated using the formulas listed in ESM Table S2. In chronological samples, the AUCs of the five, four and three miRNA combinations were 0.835, 0.829, and 0.821, respectively, which were far better than the AUC of CEA and CA19-9 (five miRNAs vs. CEA: *p* = 0.014; five miRNAs vs. CA19-9: *p* = 4.8 × 10^9^; four miRNAs vs. CEA: *p* = 0.023; four miRNAs vs. CA19-9: *p* = 8.7 × 10^9^; three miRNAs vs. CEA: *p* = 0.047; and three miRNAs vs. CA19-9: *p* = 1.1 × 10^7^) (Table 1). In contrast, there were no significant differences between these combinations (five miRNAs vs. four miRNAs: *p* = 0.18; five miRNAs vs. three miRNAs: *p* = 0.37; and four miRNAs vs. three miRNAs: *p* = 0.61) (Fig. 4d, Table 1). The chronological expression levels of these three miRNA combinations are presented in Fig. 2e (no-recurrence cases) and Fig. 3e (recurrence cases). The cut-off line for each of the three miRNA combinations is shown in Fig. 4e. The presence or absence of adjuvant chemotherapy was unrelated to this outcome.

### The Majority of Identified miRNAs are Upregulated in Primary CRC

**Fig. 5:**
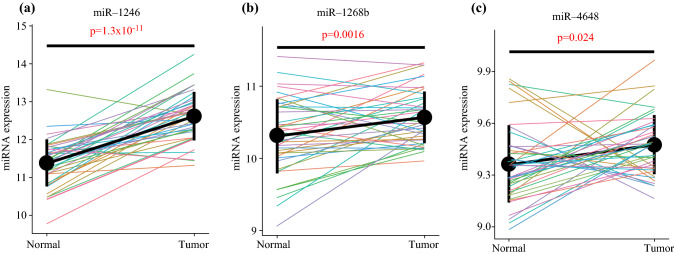
Expression of miRNAs identified in primary CRC tissue and adjacent normal tissue. **a** miR-1246; **b** miR-1268b; **c** miR-4648. Point and vertical line represents the mean with 95% confidence interval. *miRNA* microRNA, *CRC* colorectal cancer

To estimate whether the origin of these miRNAs identified in serum was in primary CRC tissue, we evaluated the expression of the 12 miRNAs in public data (GSE49246, *n* = 40).^22^ The three sets of miRNAs used in combination were significantly upregulated in primary CRC tissue compared with those in adjacent normal tissue (Fig. 5c, d, g). Therefore, we assumed that the three identified optimal miRNA combinations were derived from malignant cells. Of the 12 miRNAs, seven (miR-25-3p, miR-1246, miR-1268b, miR-2392, miR-4480, miR-4648, and miR-4732-5p) were significantly upregulated in primary CRC tissue, two (miR-451a and miR-4736) did not show a significant difference between CRC tissue and normal tissue, and three (miR-6131, miR-6776-5p, and miR-6851-5p) had no data in GSE49246 (Fig. 5 and ESM Fig. S4).

### Prediction of Target Genes of Three Identified miRNAs Used in Combination

We considered the possibility that the three miRNAs were relevant to each other and shared target genes for interpretation in a unified way. Therefore, we predicted the target genes using TargetScan^27^ (http://www.targetscan.org/vert_72/) and miRDB^28^ (http://mirdb.org/miRDB/), which were applied to predict miRNA target sites conserved among orthologous 3′ UTRs (untranslated regions) of vertebrates. In TargetScan, miR-1246 miR-1268b, and miR-4648 have 3043, 1775, and 3488 predicted target genes, respectively (data not shown), and in miRDB, miR-1246, miR-1268b, and miR-4648 have 407, 83, and 297 predicted target genes, respectively (ESM Table S3); however, these three miRNAs did not share target genes.

## Discussion

This study aimed to identify serum combinatorial miRNAs that are clinically feasible, and robust biomarkers for the early detection of CRC recurrence. Tumor-derived circulating miRNAs have been considered for oncological diagnosis since Mitchell et al. first proposed that miRNAs could be stable biomarkers for the blood-based detection of solid cancers.^29,30^ However, the most identified miRNAs do not apply to the clinical work due to the genomic and transcriptomic inter- and intra-tumor heterogeneity in CRC cases.^31,32^ In spite of the diversity issue, the former studies had been implemented with insufficient validation, a few points sampling without longitudinal observation, and a lack of cross-validation in terms of population ethnicity.^33,34^

First, we investigated whether a combination of appropriate miRNAs could surpass the diversity of CRC cases. We then selected 12 miRNAs, namely miR-25-3p, miR-451a, miR-1246, miR-1268b, miR-2392, miR-4480, miR-4648, miR-4732-5p, miR-4736, miR-6131, miR-6776-5p, and miR-6851-5p, and compared the sensitivity and specificity for incidence of CRC recurrence with conventional serum biomarkers, CEA and CA19-9. We hypothesized that the reason for the inadequate reproducibility of serum miRNAs as biomarkers is the diversity of CRC, and we expect that this diversity could be addressed by using multiple excellent miRNAs. These results indicate that the combination of optimal serum miRNAs could show adequate reproducibility as biomarkers due to the diversity of cancer. We identified candidate combinations of three serum miRNAs, miR-1246, miR-1268b, and miR-4648, which are promising clinical biomarkers of recurrence. These miRNAs are ideal combinations and yield the required ROC curves for clinical application.

As the 12 identified miRNAs were either clustered within cancer or not, the validity of our extraction method was supported. miR-451 and miR-1246 have previously been reported as biomarkers of CRC;^19,25,35^ in contrast, Niu et al. suggested miR-25-3p as part of a set of suitable reference genes in serum because there were no significant differences in its serum expression between patients with CRC and NCs; thus, the potential of miR-25-3p as a biomarker for CRC remains controversial.^36^ Correlations of the other miRNAs with CRC have not been previously reported. Additionally, several intriguing studies have recently been conducted to determine the optimal miRNA combinations. miR-1246, one of the three miRNAs we focused on, has recently gained interest as a diagnostic and prognostic marker for CRC.^19^ At maturity, miR-1246 negatively regulates the expression of *CCNG2*, which is associated with growth inhibition, and consequently promotes tumor growth and metastasis in CRC.^37^ miR-1246 has also been reported to target *CCNG2* in oral carcinomas and breast cancer.^38,39^ miR-4648 has been reported to correlate with poor prognosis in small cell carcinoma of the esophagus.^40^ miR-1268b has been reported only in gestational diabetes and dissected aortic tissue,^41^ and not in cancer. These miRNAs were derived predominantly from tumor tissues as opposed to the corresponding normal tissues. We expected that these three miRNAs would be relevant to each other and share target genes, but these miRNAs were not found to share target genes. In the TargetScan database, miR-1246, miR-1268b, and miR-4648 have 3488 predicted target genes, respectively. According to the database mid-B, miR-1246, miR-1268b, and miR-4648 have 407, 83, and 297 predicted target genes, respectively. It is unlikely that the identified miRNAs function by targeting a single gene. Using miRTargetLink 2.0^42^ (https://ccb-compute.cs.uni-saarland.de/mirtargetlink2), a tool of interactive miRNA target gene and target pathway networks, miR-25-3p has the most common target in 12 identified miRNAs (ESM Fig. S5a), indicating that miR-25-3p plays an important role in the recurrence of CRC. The shared targets between miR-25-3p and the three identified miRNAs were *CTC1* (miR-1246), *EIF5A2*, *TLN1* (miR-1268b), *CDC5L*, *VMA21*, *NLRP9* (miR-4648), and *WDR81* (miR1268b and miR-4648) [ESM Fig. S5b]. We found no evidence that these genes were involved in cancer. These results suggest that these miRNAs do not function in a unified manner, but by a complex mechanism instead.

As for the origin of miRNAs, the majority of circulating miRNAs are derived from tumor tissue.^43^ We evaluated the expression of the 12 identified miRNAs in primary CRC tissue. Therefore, the majority of the identified miRNAs were upregulated in primary CRC tissue compared with those in normal tissue, which is consistent with previous reports and supports the notion that the identified miRNAs are specific for CRC.

This study had some limitations. First, we did not consider the potential bias of hemolysis. Of the 12 identified miRNAs, miR-451 was reported as a hemolysis-susceptible miRNA.^44^ Second, the tissue and serum samples were not paired. Third, there were few cases of recurrence, and an evaluation of the usefulness of the combinations and panels in another independent large cohort is necessary. Finally, the origin and function of the identified miRNAs remain unclear.

## Conclusion

Using multiple miRNAs as serum biomarkers to represent the recurrence of CRC improves diagnostic accuracy and overcomes the diversity of CRC. The three-miRNAs, miR-1246, miR-1268b and miR-4648, could serve as optimal combinational biomarkers for CRC recurrence.

## Supplementary Information

Below is the link to the electronic supplementary material.

Supplementary file1 (PPTX 2678 kb)

Supplementary file2 (PPTX 887 kb)

Supplementary file3 (PPTX 1876 kb)

Supplementary file4 (PPTX 1451 kb)

Supplementary file5 (PPTX 1097 kb)

Supplementary file6 (DOCX 13 kb)
