# Supplementary figures and images for: Identification of the Minimum Combination of Serum microRNAs to Predict the Recurrence of Colorectal Cancer Cases
Source: Ann Surg Oncol. 2022 Sep 29;30(1):233–43. doi: 10.1245/s10434-022-12355-w (PMC9726799; doi:10.1245/s10434-022-12355-w)

## Slide 1
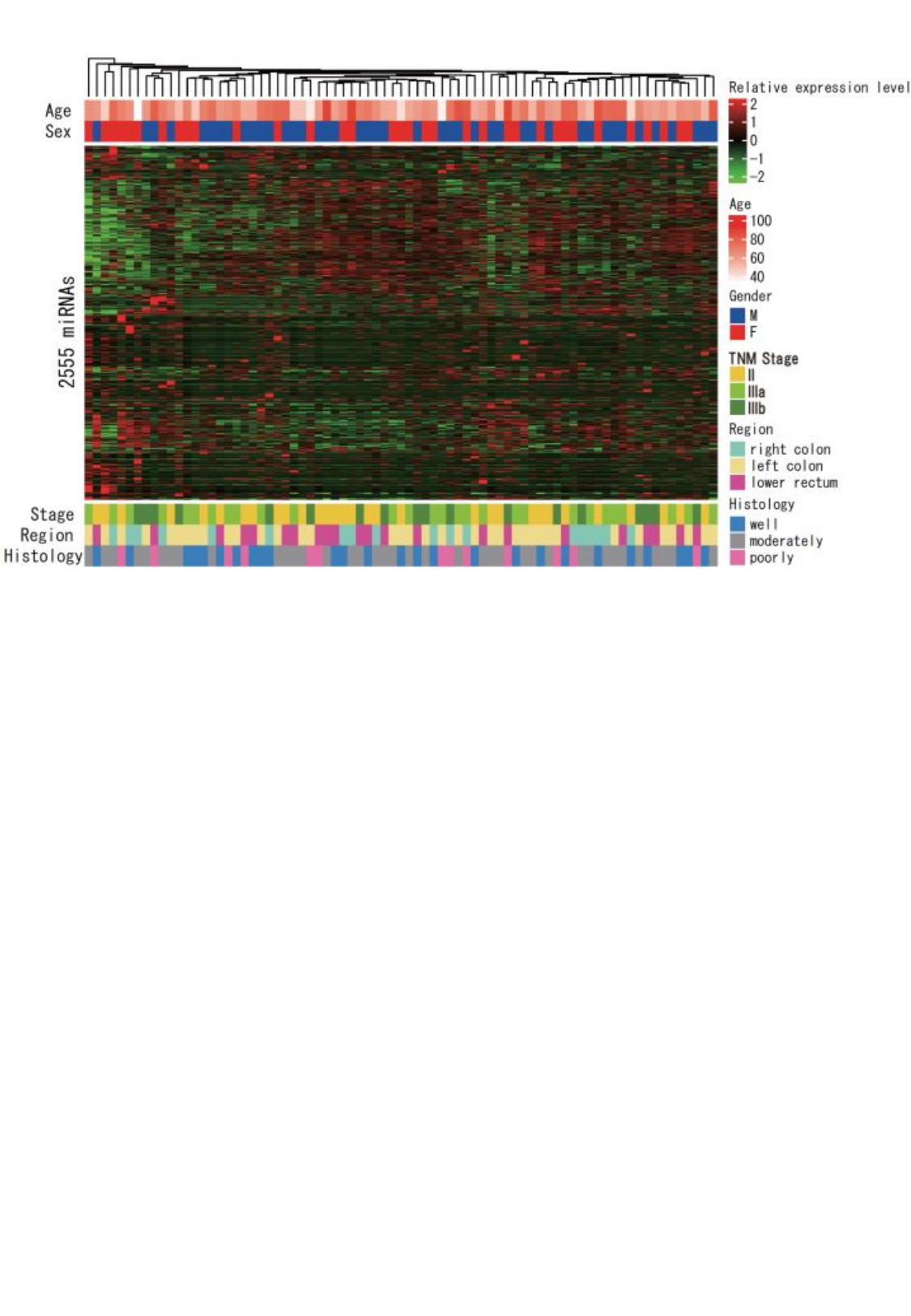

Supplement: Supplementary file 1 — Supplementary file1 (PPTX 2678 kb) [file 10434_2022_12355_MOESM1_ESM.pptx]

## Slide 1
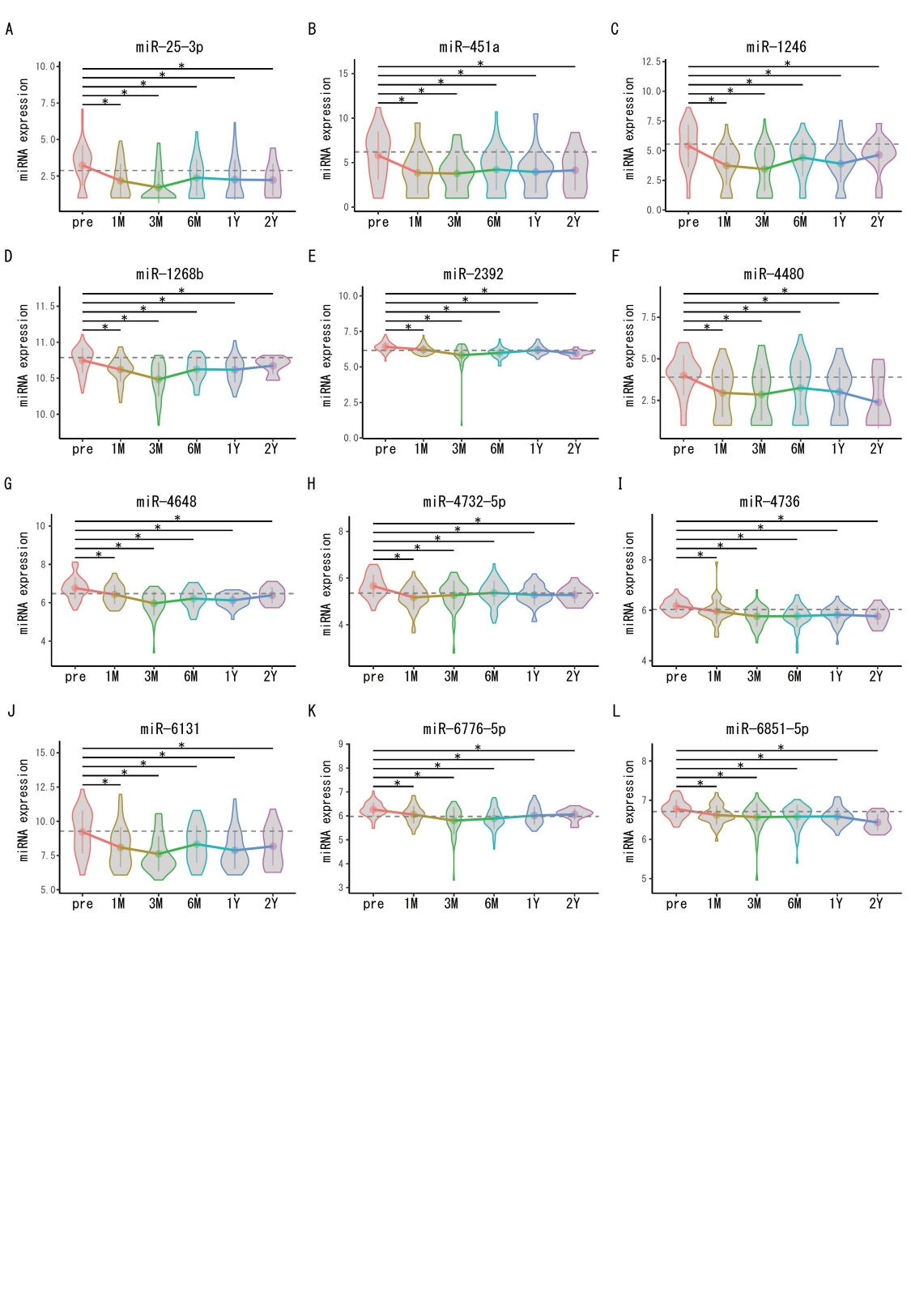

Supplement: Supplementary file 2 — Supplementary file2 (PPTX 887 kb) [file 10434_2022_12355_MOESM2_ESM.pptx]

## Slide 1
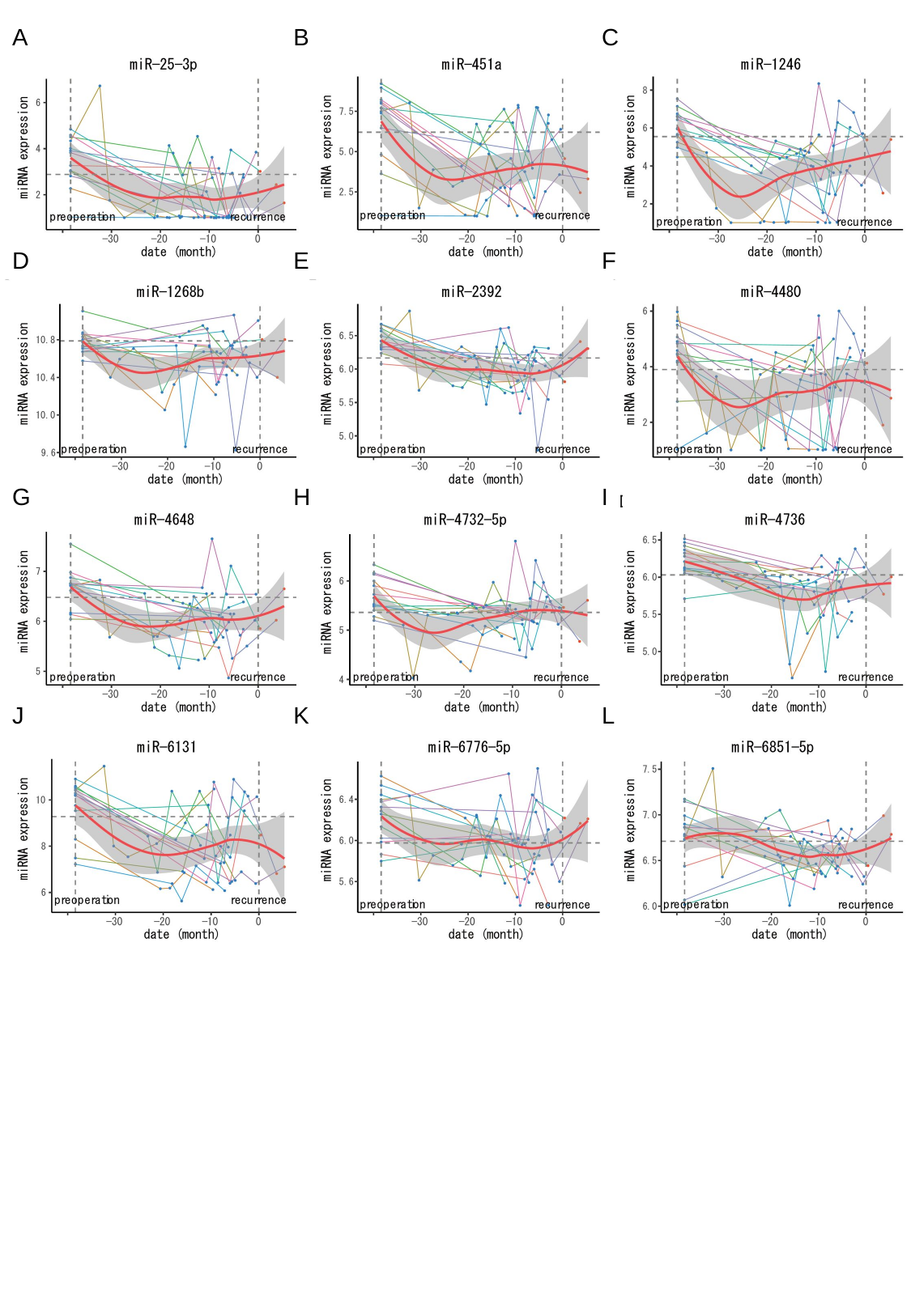

A
B
C
D
E
F
G
H
I
J
K
L

Supplement: Supplementary file 3 — Supplementary file3 (PPTX 1876 kb) [file 10434_2022_12355_MOESM3_ESM.pptx]

## Slide 1
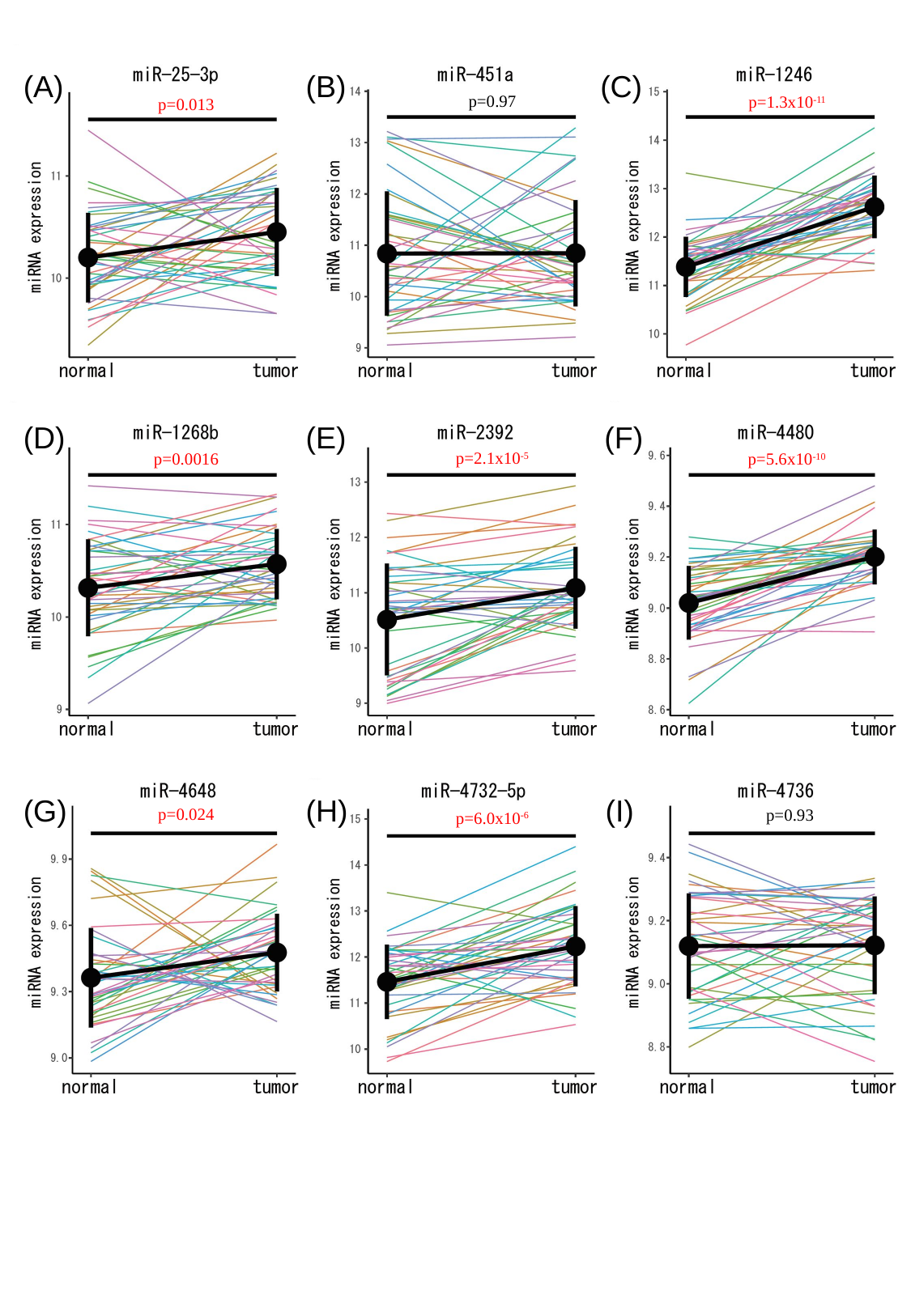

(A)
(B)
(C)
p=0.97
p=1.3x10-11
p=0.013
(D)
(E)
(F)
p=2.1x10-5
p=0.0016
p=5.6x10-10
(G)
(H)
(I)
p=0.024
p=0.93
p=6.0x10-6

Supplement: Supplementary file 4 — Supplementary file4 (PPTX 1451 kb) [file 10434_2022_12355_MOESM4_ESM.pptx]

## Slide 1
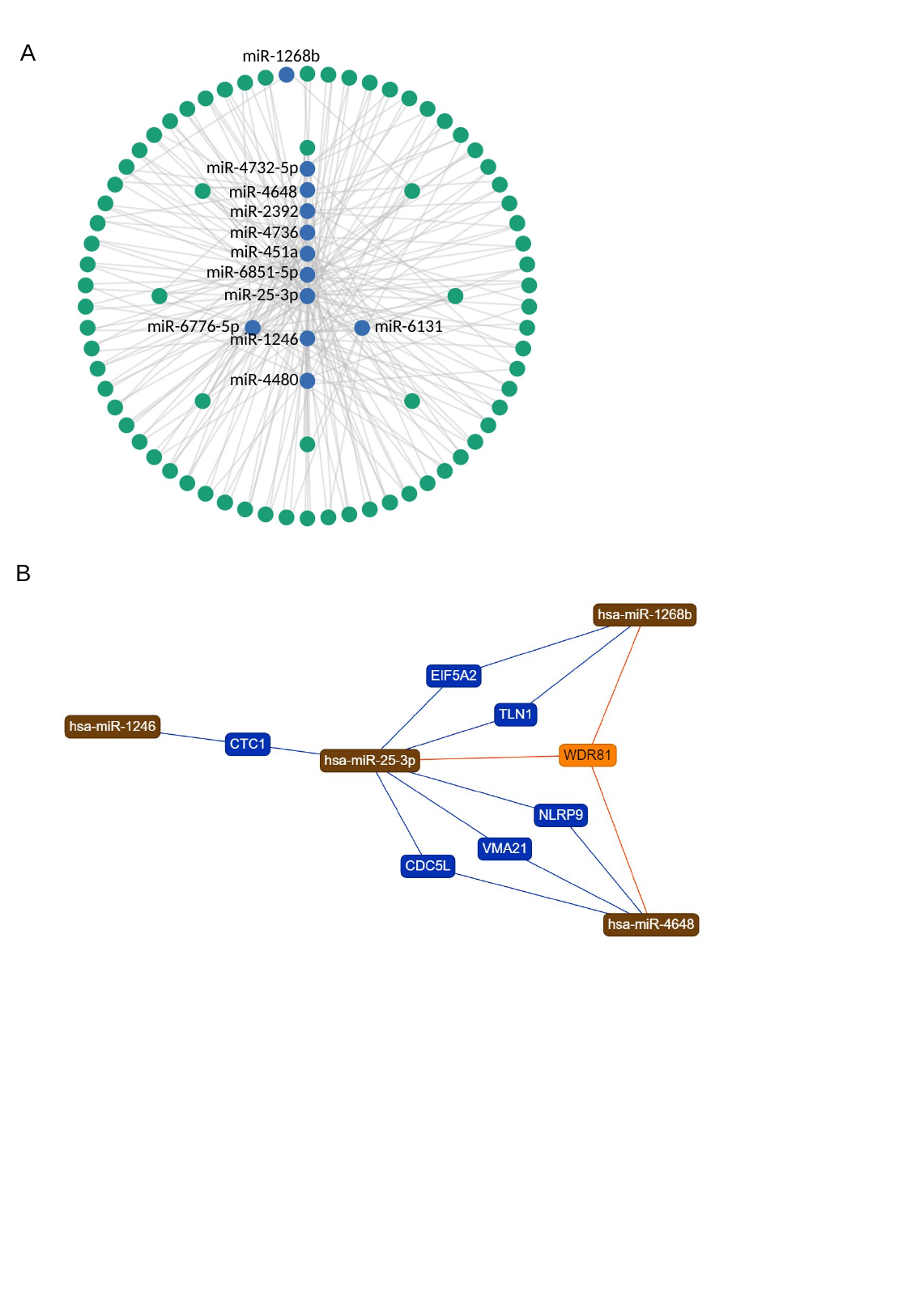

A
miR-1268b
miR-4732-5p
miR-4648
miR-2392
miR-4736
miR-451a
miR-6851-5p
miR-25-3p
miR-6776-5p
miR-6131
miR-1246
miR-4480
B

Supplement: Supplementary file 5 — Supplementary file5 (PPTX 1097 kb) [file 10434_2022_12355_MOESM5_ESM.pptx]
