# Supplementary material for: Identification of the Minimum Combination of Serum microRNAs to Predict the Recurrence of Colorectal Cancer Cases
Source: Ann Surg Oncol. 2022 Sep 29;30(1):233–43. doi: 10.1245/s10434-022-12355-w (PMC9726799; doi:10.1245/s10434-022-12355-w)
Supplement: Supplementary file 6 — Supplementary file6 (DOCX 13 kb) [file 10434_2022_12355_MOESM6_ESM.docx]

**Supplementary Table 1. Clinicopathological characteristics of 91 patients**

|  | **n = 91** |
| --- | --- |
| **Age (years)** | 65.3 ± 10.9 |
| **Sex** | M: 51, F: 40 |
| **T** | T1: 2, T2: 6, T3: 78, T4: 5 |
| **N** | N0: 39, N1: 32, N2: 12, N3: 8 |
| **Stage** | II: 39, III: 52 |
| **Region** | Right: 23, Left: 42, Lower rectum: 26 |
| **Histology** | Well: 37, Moderately: 45, Poorly: 9 |
| **Adjuvant chemotherapy** | Yes: 53, No: 18, Unknown: 20 |
| **Recurrence** | Yes: 13, No: 58, Unknown: 20 |

n: case number, T: tumour depth, N: lymph node metastasis, M: male, F: female

**Supplementary Table 2. The formulas of the diagnostic index for the miRNA combinations**

| **3 miRNAs** | (0.29 × miR-1246) + (2.16 × miR-1268b) + (0.89 × miR-4648) – 30.11 |
| --- | --- |
| **4 miRNAs** | (0.34 × miR-25-3p) + (0.21 × miR-1246) + (2.01 × miR-1268b) + (0.63 × miR-4648) – 27.44 |
| **5 miRNAs** | (0.40 × miR-25-3p) + (0.28 × miR-1246) + (2.01 × miR-1268b) + (0.66 × miR-4648) + (-0.14 × miR-6131) – 26.89 |
